# Fibroblast Growth Factor 21 Analogs for Treating Metabolic Disorders

**DOI:** 10.3389/fendo.2015.00168

**Published:** 2015-11-05

**Authors:** Jun Zhang, Yang Li

**Affiliations:** ^1^Amgen Inc., South San Francisco, CA, USA

**Keywords:** FGF21, FGF19, FGFR1, beta-Klotho, diabetes, obesity, therapy, antibody

## Abstract

Fibroblast growth factor (FGF) 21 is a member of the endocrine FGF subfamily. FGF21 expression is induced under different disease conditions, such as type 2 diabetes, obesity, chronic kidney diseases, and cardiovascular diseases, and it has a broad spectrum of functions in regulating various metabolic parameters. Many different approaches have been pursued targeting FGF21 and its receptors to develop therapeutics for treating type 2 diabetes and other aspects of metabolic conditions. In this article, we summarize some of these key approaches and highlight the potential challenges in the development of these agents.

## Introduction

Fibroblast growth factors (FGFs) are a group of signaling proteins with a conserved core domain of about 120 amino acids. These molecules display diverse functions from embryonic development, and tissue regeneration, to maintenance of metabolic homeostasis. Aberrant FGF signaling is linked to a range of pathological conditions, from cancer to metabolic disease (1, 2). FGFs function through single-pass transmembrane receptor (FGFR) proteins with three extracellular immunoglobulin-type domains (D1–D3) and an intracellular tyrosine kinase domain. Seven major FGFRs (FGFR1b, FGFR1c, FGFR2b, FGFR2c, FGFR3b, FGFR3c, and FGFR4) encoded by four *FGFR* genes mediate FGF functions. The mammalian FGF family comprises 18 members that are subdivided into six groups based on phylogenetic analysis (3). Five of the six subfamilies are paracrine molecules in that they are secreted, retained, and function in local tissues where they are produced due to high affinity interactions with heparan sulfate (HS) proteoglycans in the extracellular matrix. The exception is the FGF19 subfamily, consisting of FGF15/19, FGF21, and FGF23, which have reduced affinity for HS. Instead, they use single-pass transmembrane proteins of the Klotho family (α-Klotho or β-Klotho) as co-receptors to activate FGFR signaling in an endocrine fashion. FGF19 subfamily members play important roles in metabolic pathways including bile acid metabolism, energy expenditure, and glucose, lipid, vitamin D, and phosphate homeostasis (3).

The human FGF21 is a 209 amino acid protein with a mature secreted polypeptide of 181 amino acids. FGF21 is mainly expressed in the liver, but is also upregulated in many other tissues upon various stimulations (4, 5). While it binds to FGFRs with very low affinity in the presence of heparin/HS, FGF21 efficiently binds to and activates FGFR1c, FGFR2c, and FGFR3c in the presence of co-receptor β-Klotho *in vitro* (6–8). *In vivo*, β-Klotho and FGFRs are co-expressed in several tissues in which FGF21 is shown to signal, and thus are potential target tissues mediating its physiological and pharmaceutical actions.

Many studies reveal FGF21 as a multifunctional metabolic regulator and its levels are significantly increased under many pathological conditions. For example, in mice, studies from *Fgf21* knockout (KO) and transgenic mice suggest diverse actions of FGF21 on many metabolic organs to regulate glucose, lipid, and energy metabolisms (9–12). In humans, circulating FGF21 levels are elevated in obese subjects (13, 14), patients with impaired glucose tolerance (15), type 2 diabetes mellitus (13, 15, 16), dyslipidemia (17), and non-alcoholic fatty liver disease (NAFLD) (18, 19). Elevated serum FGF21 levels are also seen in subjects with coronary heart disease (CHD) (20, 21) and in patients with carotid atherosclerosis (22). Serum FGF21 levels increase with progression of chronic kidney disease (CKD), chronic and acute renal dysfunction (23, 24), cold-induced thermogenesis, and brown adipose tissue (BAT) activity (25, 26). Altogether, these findings suggest that targeting FGF21 and its pathway components may have broad impact on various human conditions. Since the physiological and pharmacological functions of FGF21 have been extensively reviewed in Ref. (27–34), in this article, we only briefly summarize the potential connections between FGF21 and disease conditions to suggest potential areas that may benefit from FGF21 treatment. We will focus the discussion on key approaches for therapeutic development of FGF21 analogs.

## FGF21 Signaling in Metabolic Diseases

### Role of FGF21 in Diabetes and Obesity

FGF21 has emerged as an important metabolic hormone involved in the regulation of glucose, lipid, and energy homeostasis. FGF21 transgenic animals show lower levels of serum insulin, glucose, triglycerides (TG), cholesterol, lower hepatic TG content, improved insulin sensitivity, resistance to diet-induced obesity, and a significantly extended lifespan compared with their wild-type counterparts (35–37). Likewise, recombinant FGF21 also demonstrates a dramatic effect in normalizing plasma glucose levels, improving insulin sensitivity, reducing plasma TG and cholesterol levels in various diabetic animal models and diabetic rhesus monkeys, exhibiting an attractive profile as a potential novel therapy for treating metabolic disorders (35, 38–42).

The ability of FGF21 to affect a major metabolic tissue function was first demonstrated on adipocytes. FGF21 increases insulin-independent glucose uptake into cultured 3T3-L1 mouse adipocytes and primary human adipocytes through the upregulation of glucose transporter 1 (GLUT1) expression (35). Subsequent studies reveal that FGF21 regulates adipocyte lipolysis (5, 43, 44) and increases adiponectin expression and secretion (45, 46). The central role of fat tissue to FGF21 function was demonstrated in a lipoatrophic mouse model (mice severely depleted of adipose tissue) (47), where recombinant FGF21 treatment cannot correct the hyperglycemia and insulin resistance in these mice in contrast to wild-type animals. Reconstitution of adipose mass by transplanting white adipose tissue (WAT) from wild-type mice to lipoatrophic mice restores FGF21 responsiveness (48). Fat-specific KO of FGF21 receptors, β-Klotho and FGFR1, abolishes FGF21-induced bodyweight reduction and improvement in serum glucose and lipid parameters (36, 49, 50), further suggesting an important role for fat tissue in FGF21 biology.

FGF21 stimulates uncoupling protein 1 (UCP1) gene expression in both BAT and WAT and dramatically increases the appearance of “brown-like” adipocytes in subcutaneous WAT (51–53). These effects may contribute to the thermogenic activities of FGF21 and to promote weight loss under high fat conditions. In mice, FGF21 expression is upregulated in response to cold exposure and β-adrenergic stimulation (11, 54, 55). In rats, cold exposure induces a marked release of FGF21 from BAT, suggesting an endocrine role of BAT as a source of FGF21 that may be relevant in thermogenic activation (55). In humans, changes in serum FGF21 concentrations correlate positively with cold-induced thermogenesis (25), and circulating FGF21 levels correlate with BAT activity during acute cold exposure in male subjects, indicating a possible role for FGF21 in maintaining normothermia (26). Although two recent studies utilizing *Ucp1* KO mice challenge the importance of WAT browning in FGF21 mediated pharmacology, the importance of these effects in humans remain to be resolved (56, 57).

Brain is another direct target of FGF21 action. FGF21 crosses the blood–brain barrier, and FGFRs and β-Klotho are expressed in certain areas of the brain [reviewed in Ref. (34)]. Central administration of FGF21 increases energy expenditure, insulin sensitivity, and hepatic gluconeogenesis, while knocking out β-Klotho centrally abolishes the effects of FGF21 on metabolism, circadian behavior, growth, and female reproduction (34), demonstrating an essential role of the nervous system in the actions of FGF21. Future studies are needed to understand the interplay between brain and adipose tissue in FGF21 responses.

### Role of FGF21 in Liver, Chronic Kidney, and Cardiovascular Diseases

Liver is a major site for both production and action of FGF21. The hepatic expression and plasma levels of FGF21 in mice are markedly elevated upon fasting, or consumption of a ketogenic diet, and are suppressed by re-feeding (5, 58, 59). It is induced directly by peroxisome proliferator-activated receptor (PPAR) α activation and mediates hepatic ketogenesis, and increases fatty acid oxidation, glucose flux, gluconeogenesis, and improves insulin sensitivity (5, 39, 40, 58, 59). Overexpression of FGF21 or recombinant FGF21 injection exhibit increased ketogenesis in liver (5) and *Fgf21* KO mice show impaired fatty acid oxidation in liver and develop hepatosteatosis (11). Elevated FGF21 levels have been associated with multiple liver diseases, including alcoholic liver diseases (ALD), NAFLDs, non-alcoholic steatohepatitis (NASH), hepatocellular carcinoma (HCC), and hepatitis, although the role of FGF21 in the progression of these diseases has not been defined (60).

Understanding the direct function of FGF21 on liver is complicated since most studies have focused on NAFLD in the context of obesity and pharmacologic doses of FGF21 induce rapid weight loss, making it difficult to identify the primary effects of FGF21 on liver metabolism. To bypass weight loss-associated benefits, a lean model of fatty liver disease was induced by consumption of a methionine- and choline-deficient (MCD) diet (61). This model recapitulates most pathologic processes observed in fatty liver disease, with progression to NASH and development of severe inflammation and fibrosis, without weight gain or insulin resistance. Mice fed with the MCD diet have more than 50-fold increased hepatic levels of FGF21 messenger RNA and 16-fold increased serum FGF21 levels. In *Fgf21* KO mice, MCD diet induces more severe steatosis, fibrosis, inflammation, and peroxidative damage. These animals show reduced hepatic fatty acid activation and β-oxidation, resulting in increased levels of free fatty acid. Continuous subcutaneous infusion of recombinant FGF21 for 4 weeks in *Fgf21* KO animals reverse steatosis and peroxidative damage, suggesting a direct and perhaps adipose independent role of FGF21 in liver fatty acid activation and oxidation (61).

Kidney is another tissue affected by FGF21 function. Serum FGF21 levels increase with the progression of CKD, and with chronic and acute renal dysfunction (23, 24). In patients with diabetic nephropathy, FGF21 levels are also independently associated with urinary albumin excretion (62), although studies on direct renal effects of FGF21 remain limited. In a recent report, recombinant FGF21, injected daily for 12 weeks into leptin receptor KO *db/db* mice, markedly decreases urinary albumin excretion and mesangial expansion and suppresses the synthesis of profibrotic molecules (63). Furthermore, FGF21 administration decreases cholesterol and triglyceride accumulation in the diabetic kidney, and markedly abolishes the increase of p65 protein in the proinflammatory NF-κB pathway, all contributing factors to the progression of diabetic nephropathy (63).

Elevated serum FGF21 levels are also observed in patients with CHD (21, 64) and associate with both carotid atherosclerosis in women and carotid artery plaques in type 2 diabetes subjects (22, 65). Furthermore, serum FGF21 levels positively correlate with carotid and iliac lesions in patients with subclinical atherosclerosis, especially in women. High levels of FGF21 may be a compensatory reaction to offset atherosclerosis (66).

Several recent reports examined the role of FGF21 in cardiovascular diseases. FGF21 is produced by cardiomyocytes (12). Although levels of FGF21 secreted from the heart is much lower than from liver, FGF21 secreted by cardiac cells in response to cardiac stress is able to inhibit isoproterenol-induced cardiac hypertrophic damages, and protects cardiac cells against hypertrophic insults (12). The increased heart weight, signs of dilatation and cardiac dysfunction in response to isoproterenol infusion are exacerbated in *Fgf21* KO animals. Treatment with recombinant FGF21 reverses cardiac hypertrophy in *Fgf21* KO mice and cultured cardiomyocytes. This paracrine action of FGF21 on cardiomyocytes prevents cardiac hypertrophy by activating MAPK signaling, inducing rapid phosphorylation of cAMP response element-binding protein (CREB), leading to increased expression of PGC-1α and repressed NF-κB proinflammatory pathway (12). FGF21 treatment also induces the expression of genes from antioxidative pathways, reduces reactive oxygen species production, and protects cells against oxidative stress, improving overall heart function and prevents heart failure (67). In a mouse model with severe defective TG catabolism due to deficiencies in adipose triglyceride lipase (ATGL), a drastic increase in heart FGF21 mRNA expression is observed. In cardiac-specific over-expression of FGF21 (CM-Fgf21) animals, FGF21, secreted from cardiomyocytes, moderately affects cardiac TG homeostasis, indicating that FGF21 is induced upon cardiac ER stress and regulates cardiac as well as whole body energy homeostasis (68). All of these findings together suggest that modulating FGF21 pathway may have broad benefits on multiple human conditions.

The elevated FGF21 levels observed under many disease conditions may indicate a state of FGF21-resistance as suggested by several studies (64, 69). However, alternative explanations, for example, that the FGF21 in diseased conditions represents less active form of the hormone due to modifications, are also possible. Nonetheless, the ability to respond to pharmacological doses of FGF21 despite the increased levels under disease conditions supports the notion that FGF21 treatment may be beneficial in these disease areas.

## Therapeutic Approches Tarteting FGF21 Pathway

The impressive beneficial effects exhibited by FGF21 treatment in various animal models on multiple metabolic parameters prompted considerable interest in developing therapeutics from this pathway. However, the native FGF21 molecule is not suitable as a development candidate due to short plasma half-life and poor drug-like properties (32). Considerable engineering efforts are needed to bring sufficient improvements for large-scale manufacturing and further clinical development. This section highlights several key approaches explored in the search of a suitable therapeutic candidate.

### Engineering the Native FGF21 Molecule

Extensive protein engineering on FGF21 itself yields significant improvements in biophysical properties in several different expressions systems. For example, the introduction of a disulfide bond at L118C–A134C in the C-terminal domain of FGF21, site-specific mutagenesis at S167 to A, and the deletion of the first four amino acids, HPIP, are found to improve the stability, and reduce the heterogeneity and proteolysis issues associated with the wild-type protein in the *Pichia pastoris* yeast expression system (70). A molecule, LY2405319, that combined all three changes together, is indistinguishable from native FGF21 in physiological effects both *in vitro* and in preclinical rodent and monkey models, but with improved biophysical properties suitable for large-scale production (70, 71). LY2405319 is the first FGF21 analog to enter human clinical trial, a phase I study was conducted in obese subjects with type 2 diabetes (70). Overall, the clinical data are similar to the pharmacologic effects of FGF21 in rodent and non-human primates, LY2405319 significantly lowers body weight, fasting insulin levels, fasting TG, total and LDL-cholesterol, and robustly increases serum adiponectin and HDL-cholesterol levels. However, only a trend toward glucose lowering is observed (70). These results are encouraging and supports that FGF21 is bioactive in humans; however, the effectiveness of FGF21 in controlling hyperglycemia would still need to be addressed in future studies.

Engineering efforts for other expression systems are also described. To efficiently produce FGF21 from *Escherichia coli*, Ye et al. exchanged the beta10-beta12 domain of human FGF21 with that of the mouse sequence and then fused the chimeric protein to SUMO (72). The solubility of the resulting SUMO-FGF21 protein is improved by twofold compared with the wild-type human FGF21. The engineered FGF21 is also more potent in stimulating glucose uptake in HepG2 cells and shows improved glucose-lowering effects in diabetic *db/db* mice (72).

Other alterations are also explored to improve the stability and bioactivity of FGF21. For example, the C-terminal domain of FGF21 crucial for interaction with co-receptor β-Klotho (73–75) is also sensitive to proteolysis (76). Replacement of this segment with sequences that are both resistant to proteolytic cleavage and have higher affinity for β-Klotho can achieve both of the goals of improving stability and bioactivity. In one approach, this C-terminal domain of FGF21 is replaced with that of FGF19, believed to bind to β-Klotho with higher affinity (77); while in another approach, this C-terminal domain is replaced with a stable scaffold called Avimer™ that has high affinity to co-receptor (78). The avimer fusion molecule, FGF21 (1-171)-WD22, indeed has improved properties and decreased susceptibility to modifications compared to wild-type FGF21 (78), providing a novel framework for further engineering that is not possible with the wild-type protein.

To extend FGF21 plasma half-life by reducing clearance through kidney filtration, several size-extension approaches, such as PEGylation, fusion to Fc, serum albumin, or full antibodies, are all reported to improve the pharmacokinetic properties of FGF21.

PEGylation to FGF21 can be achieved through the N-terminal residue or a specific site on the surface of the protein. N-terminal PEGylation using 20 kDa mPEG-butyraldehyde extends FGF21 plasma half-life from ~35 min to ~4 h with reduced immunogenicity (79). Internal PEGylation can be carried out by mutating specific amino acid residues for attachment to a cysteine residue (80) or to a non-natural amino acid, p-acetylphenylalanine (81), followed by conjugation reactions for PEG attachment. In these cases, the plasma half-life extension achieves >30 h in rodent. In general, PEGylation at certain positions retains good FGF21 activities *in vitro* and *in vivo* (79–81). In addition, correctly placed PEG moieties also reduce the risk of inducing kidney vacuole formation (80).

Alternative to PEGylation, half-life extension via fusion to Fc molecules has the potential advantage of reducing manufacturing complexity and improving homogeneity of the final product. A FGF21 analog based on fusion to the Fc fragment of human IgG1 reports such improved properties (76). The final molecule, Fc-FGF21(RG), consists of the combination of several changes, these include the selection of the N-terminus of FGF21 for Fc fusion to reduce impact on receptor binding, mutation at P171 to G to remove a proteolytic clipping site, and substitution of L98 to R to reduce aggregation (76). Fc-FGF21(RG) reports a circulating half-life of 11 h in mice and 30 h in monkeys (76, 82). In rodent models and obese rhesus monkeys, a single injection of Fc-FGF21(RG) displays a sustained reduction in blood glucose levels, lipids, and bodyweight for 5–7 days, demonstrating a profile consistent with once-weekly dosing (83).

Fusion to scaffold monoclonal antibody also proves successful in extending FGF21 plasma half-life (84). A mutated FGF21 molecule is conjugated to the Fab region of a scaffold antibody, CVX-200, via one of the four native lysine residues (K56, K59, K69, or K122) or one of the three positions mutated to cysteine (D79C, H125C, and A129C). One such conjugated molecule, CVX-343, which is the fusion of the FGF21-A129C mutant to the scaffold antibody retains *in vitro* activity while increasing half-life up to 70-fold compared with wild-type FGF21. In diet-induced obese mice, weekly doses of CVX-343 reduce body weight, blood glucose, and lipid levels. In *ob/ob* and *db/db* mice, CVX-343 increases glucose tolerance, pancreatic beta-cell mass and proliferation, providing evidence of another half-life extended FGF21 molecule with improved preclinical pharmacokinetics while preserving full therapeutic functionality.

### Other FGF-Based Analogs

Several other FGF molecules show similar effects on metabolism to FGF21 (32, 85). In addition, advances in understanding FGF/receptor interactions/functions offer alternative approaches to engineer FGF21-like or mimetic molecules. So far, FGF19, FGF23, FGF1, and FGF2 have been explored for this concept.

The most advanced analog is based on FGF19. FGF21 and FGF19 share an extensive receptor binding profile and pharmacological effects [reviewed in Ref. (32, 86)]. Both bind β-Klotho, and both activate β-Klotho complexed with FGFR1c, 2c, and 3c. Both FGF19 and FGF21 display a similar metabolic profile, such as lowering serum glucose, TG and cholesterol levels, as well as improving insulin sensitivity and reducing bodyweight in multiple disease models, suggesting that FGF19 could be an alternative to FGF21 for therapeutic development. However, the key concern for FGF19 is that in addition to FGFR1c, 2c, and 3c, FGF19 also regulates bile acid homeostasis and induces hepatocyte proliferation and HCC via activation of FGFR4 (51, 87–89). Therefore, selective removal of mitogenic activity is a strategy for FGF19 development. FGF19 variants devoid of the undesirable proliferative activities have been identified (88, 90–92) and one variant has been tested in humans (92), the efficacy and long-term safety profiles in humans remain to be established.

A similar strategy also proved successful for FGF1. FGF1, considered a prototypic FGF molecule discovered in the early 1970s, has also been shown recently to play a role in adaptive adipose remodeling and metabolism (85, 93). Similar to FGF19, the mitogenic and metabolic activities of FGF1 are separable. A variant of FGF1, devoid of mitogenic activity, improves glucose metabolism and insulin sensitivity in a rodent model, demonstrating a potential utility of FGF1 in treating diabetes in humans (85).

Structural and functional studies of various FGF molecules suggest another interesting approach where the core FGF sequence can be considered as a protein scaffold, and sequences outside the core can be considered as appendages enabling a specific biological activity through specific interactions with FGFRs, co-receptors, and/or heparin (90). This opens the possibility to explore the entire FGF family for designing the best candidate for development (90). Two successful examples are reported, one with endocrine factor, FGF23 (94), the other with a paracrine factor, FGF2 (95). In the case of FGF23, since it also activates FGFR1c, 2c, and 3c (94), but differs from FGF21 in co-receptor utilization (6, 96, 97), the replacement of the α-Klotho interacting FGF23 C-terminal domain with the β-Klotho interacting FGF21 C-terminal domain converts FGF23 into an FGF21-like molecule (94). In the case of FGF2, in addition to adding the β-Klotho interacting C-terminal region of FGF21 (P168–S209) to the FGF2 framework from M1 to M151, mutations in the HS-binding site (K128D/R129Q/K134V) are also needed to reduce heparin binding. The resulting molecule, FGF2^ΔHBScore^-FGF21^C-tail^, loses heparin binding and HS-dependent activation of FGFR1c, but gains the ability to function like FGF21 to regulate metabolism (95).

### Non-FGF-Based Analogs

While significant improvements have been made to FGF21 and other FGF-based FGF21 analogs, additional challenges may need to be addressed as the molecules progress through development. For example, potential induction of anti-drug antibodies that neutralize endogenous ligand function may pose risk in the clinic. Therefore, protein scaffolds or modalities that have more optimal drug-like properties, but do not have sequence homology to endogenous ligands, may circumvent challenges associated with the use of endogenous ligands themselves. Since FGF21’s pharmacological function is mainly mediated through FGFR1c/β-Klotho activation [reviewed in Ref. (32)], several innovative approaches using monoclonal antibodies (mAbs) and other stable scaffolds to target components of the receptor complex have been reported.

A subset of mAbs, identified through phage display screening that selectively bind both FGFR1 isotypes, are found to be agonistic and able to activate receptor signaling (98, 99). In multiple diabetic animal models, the injection of these FGFR1 agonistic antibodies induce acute and sustained serum glucose lowering, decrease serum insulin levels and bodyweight, and improve lipid profiles and hepatosteatosis. In fat tissue, FGF21 and anti-FGFR1 antibodies induce CREB phosphorylation and increase expression of PGC-1α, PGC-1β, UCP-1 and the downstream genes associated with oxidative metabolism. Overall, the effects of these agonist antibodies mimic FGF21 actions. Since these antibodies do not depend on β-Klotho to signal, the non-tissue-specific activation of FGFR1 also induces undesirable side effects. For example, these antibodies induce FGF23 production and hypophosphatemia owing to their actions in bone and kidney, and potential ability to induce proliferation may be another concern (98, 100).

An antibody, mimAb1 identified through immunization of XenoMouse, specifically activates FGFR1c and is completely β-Klotho dependent (49). MimAb1 displays similar potency and efficacy in inducing ERK phosphorylation *in vitro* when compared with recombinant FGF21. In obese cynomolgus monkeys, mimAb1 injections result in reductions in bodyweight, plasma insulin and TG, and improvement in insulin sensitivity similar to Fc-FGF21, but with significantly extended duration of effect (49).

FGFR1/β-Klotho specificity and β-Klotho dependency is also achieved through another approach, the generation of a bispecific molecule where one arm binds β-Klotho while the other binds FGFR1. This concept was first demonstrated using the Avimer™ scaffold. A-domain library is screened for Avimer™ polypeptides that bind FGFR1 or β-Klotho (101). The individual binders are then assembled into a single polypeptide, generating a bispecific molecule that binds to β-Klotho and FGFR1c simultaneously. One such bispecific avimer, C3201, is highly potent in FGF21-sensitive cell-based assays, activates FGFR1c signaling with efficacy similar to FGF21 and depends on β-Klotho. To extend its serum half-life, C3201 is fused to human serum albumin (HSA), and the resulting fusion molecule, C3201-HSA, exhibits a half-life sufficient for once-weekly dosing. When tested in male obese cynomolgus monkeys, animals treated with C3201-HSA show improved metabolic parameters with reduced bodyweight, insulin, and TG levels similar to FGF21 (101). Therefore, these data demonstrate the concept that a bispecific molecule, generated by fusing a FGFR1 binder and a β-Klotho binder together, can activate appropriate receptor signaling and mimic the activity of FGF21 *in vitro* and *in vivo*. Generation of such a bispecific molecule is not restricted to an Avimer scaffold, other scaffolds can also be used. A variation of this approach using a bispecific antibody has also been shown to be successful. FGFR1 binders and β-Klotho binders, obtained through phage display and immunization, respectively, are assembled using the knob and hole method (102). The resulting bispecific molecules resemble a normal antibody conformation except with the two arms capable of binding FGFR1 and β-Klotho separately. Similar to bispecific Avimers, these bispecific antibodies also display activities similar to FGF21, stimulate thermogenic activity in BAT, induce WAT browning, and ameliorate obesity, insulin resistance, and associated metabolic defects. Unlike the antibodies activate FGFR1 independent of β-Klotho, the bispecific antibodies do not alter serum FGF23 and phosphorus levels (98, 100, 102), supporting the notion that increase target specificity through β-Klotho dependency reduces undesirable side effects of FGFR activation.

## Summary

An FGF21 therapeutic represents an attractive opportunity for novel drug development treating metabolic disorders. Many of its potent activities in improving glucose metabolism, lipid metabolism, inducing energy expenditure and reducing bodyweight, demonstrated in many rodent and non-human primate models, are translated to humans.

Various approaches successfully improve FGF21 stability, reduce aggregation, improve affinity toward receptor, and improve its manufacturability and productivity (Table 1). Additionally, FGF21-mimetic molecules utilizing completely distinct protein modalities, with no similarities to endogenous ligand, such as agonistic antibodies and bispecific Avimers, open new opportunities to identify best-in-class therapeutics for this pathway (Table 1). However, significant challenges also exist. Although initial clinical data provide encouraging signs of efficacy in humans, it is unclear if FGF21 could provide clinically meaningful improvements in hyperglycemia or more robust and durable responses in other metabolic parameters under chronic treatment conditions. In addition, studies in rodents with FGF21 reveal several potential areas of side-effect concerns that need monitoring, such as potential interactions with the growth hormone axis (103, 104), decreases in bone mineral density (105), increases in plasma corticosterone levels (106), cross-talk with the PPARγ axis (107), and female infertility (108). The human relevance of these rodent findings, as well as the potential differences in side-effect profiles among the different therapeutic approaches, will need to be evaluated in the future.

**Table 1:** **Selected list of FGF21 analogs**.

Target	Compound (source)	Reference
FGF21 analogs	LY2405319 (Eli Lilly)	(70)
	SUMO-FGF21 (NAU)	(72)
	FGF21/19 chimera (NYU)	(77)
	FGF21(1-171)-WD22 (Amgen)	(78)
FGF21 long-acting analogs	PEG-FGF21 (WMC)	(79)
	PEG-FGF21 (Amgen)	(80)
	ARX-618 (Ambrx/BMS)	(81, 109)
	Fc-FGF21 (Amgen)	(76)
	CVX-343 (Pfizer)	(84)
Other FGF-based analogs	FGF19 variants (Amgen, Genentech, NGM)	(88, 90–92)
	FGF23-21 (Amgen)	(94)
	rFGF1^ΔNT^ (Salk/NYU)	(85)
	FGF2^ΔHBScore^-FGF21^C-tail^ (NYU)	(95)
Non-FGF-based analogs	R1Mab (Genentech)	(98)
	mimAb1 (Amgen)	(49)
	C3201-HSA (Amgen)	(101)
	bFKB1 (Genentech)	(102)

## Conflict of Interest Statement

Both authors are full-time employees of Amgen Inc.
